# Association between mitral valve prolapse and sudden sensorineural hearing loss: A case-control population-based study

**DOI:** 10.1371/journal.pone.0205199

**Published:** 2018-10-04

**Authors:** Yen-Fu Cheng, Tzong-Han Yang, Chuan-Song Wu, Chung-Chien Huang, Herng-Ching Lin

**Affiliations:** 1 Department of Medical Research, Taipei Veterans General Hospital, Taipei, Taiwan; 2 Department of Otolaryngology-Head and Neck Surgery, Taipei Veterans General Hospital, Taipei, Taiwan; 3 Department of Otolaryngology-Head and Neck Surgery, School of Medicine, National Yang-Ming University, Taipei, Taiwan; 4 Department of Speech, Language and Audiology, National Taipei University of Nursing and Health Sciences, Taipei, Taiwan; 5 Research Center of Sleep Medicine, College of Medicine, Taipei Medical University, Taipei, Taiwan; 6 Department of Otolaryngology, Taipei City Hospital, Taipei, Taiwan; 7 School of Health Care Administration, College of Management, Taipei Medical University, Taipei, Taiwan; Universidade Federal de Sao Paulo, BRAZIL

## Abstract

This study aimed to evaluate the relationship between sudden sensorineural hearing loss (SSNHL) and MVP using a population-based dataset. Data for this case-control study were retrieved from the Taiwan Longitudinal Health Insurance Database. In total, 3399 cases of newly diagnosed SSNHL were identified. We used propensity score matching to select 3399 comparison patients (one for every case) from the same dataset. The selected matching variables included age, sex, monthly income, geographical location, urbanization level of the patient’s residence, hypertension, diabetes, and hyperlipidemia. Chi-squared tests were used to compare differences in sociodemographic characteristics, while conditional logistic regression analyses were used to examine the association of SSNHL with previously diagnosed MVP. Of the 6798 sampled subjects, 131 (1.93%) patients had received a diagnosis of MVP prior to the index date. A significant difference in the prevalence of prior MVP between cases and controls (2.41% vs. 1.44%, *p =* 0.004) was observed. The conditional logistic regression analysis conditioned on gender, age, monthly income, urbanization level, geographic region, hyperlipidemia, diabetes, and hypertension suggested that the odds ratio of prior MVP for cases was 1.69 (95% confidence interval (CI): 1.18~2.42) compared to controls. Our study found that patients with MVP had a 1.69-fold higher risk of getting SSNHL compared to patients without MVP.

## Introduction

Mitral valve disease (MVP) is a moderately common valvular abnormality that features displacement of the mitral valve apparatus into the left atrium toward the end of systole[1, 2]. MVP is clinically defined as systolic override of one or both mitral leaflets of ≥2 mm above the mitral annulus plane in the parasternal long axis or apical three-chamber view[3]. MVP is the most prevalent mitral valve disorder, affecting approximately 2.4% of the general population. MVP is infrequent among children but is commonly found in adults, who present with heart palpitations, fatigability, chest pain, and dyspnea.

The association of MVP with sudden sensorineural hearing loss (SSNHL) was first documented in case reports by Moulonguet et al., although its pathogenesis remains unclear[4]. Vazquez et al. conducted a prospective controlled study to investigate the prevalence of MVP among patients with SSNHL and found a higher incidence[5]. These various studies therefore implied cardiovascular compromise in SSNHL that very likely originates from MVP.

However, to the best of our knowledge, the risk between the two entities has never been directly compared to that of the general population. Hence, this study aimed to explore the relationship between MVP and SSNHL using a nationwide population-based dataset.

## Materials and methods

### Database

We retrieved the study sample for this case-control study from the Longitudinal Health Insurance Database 2005 (LHID2005). The LHID2005 consists of medical claims data of 1,000,000 individuals randomly sampled from the 2005 Registry for Beneficiaries (*n* = 25.68 million) of the Taiwanese National Health Insurance (NHI) program. The LHID2005 allows researchers in Taiwan to trace all enrollees’ use of medical services since the beginning of the Taiwanese NHI program in 1995. This study was approved by the institutional review board of Taipei Medical University (TMU-JIRB N201708046).

### Selection of cases and controls

**Fig 1:**
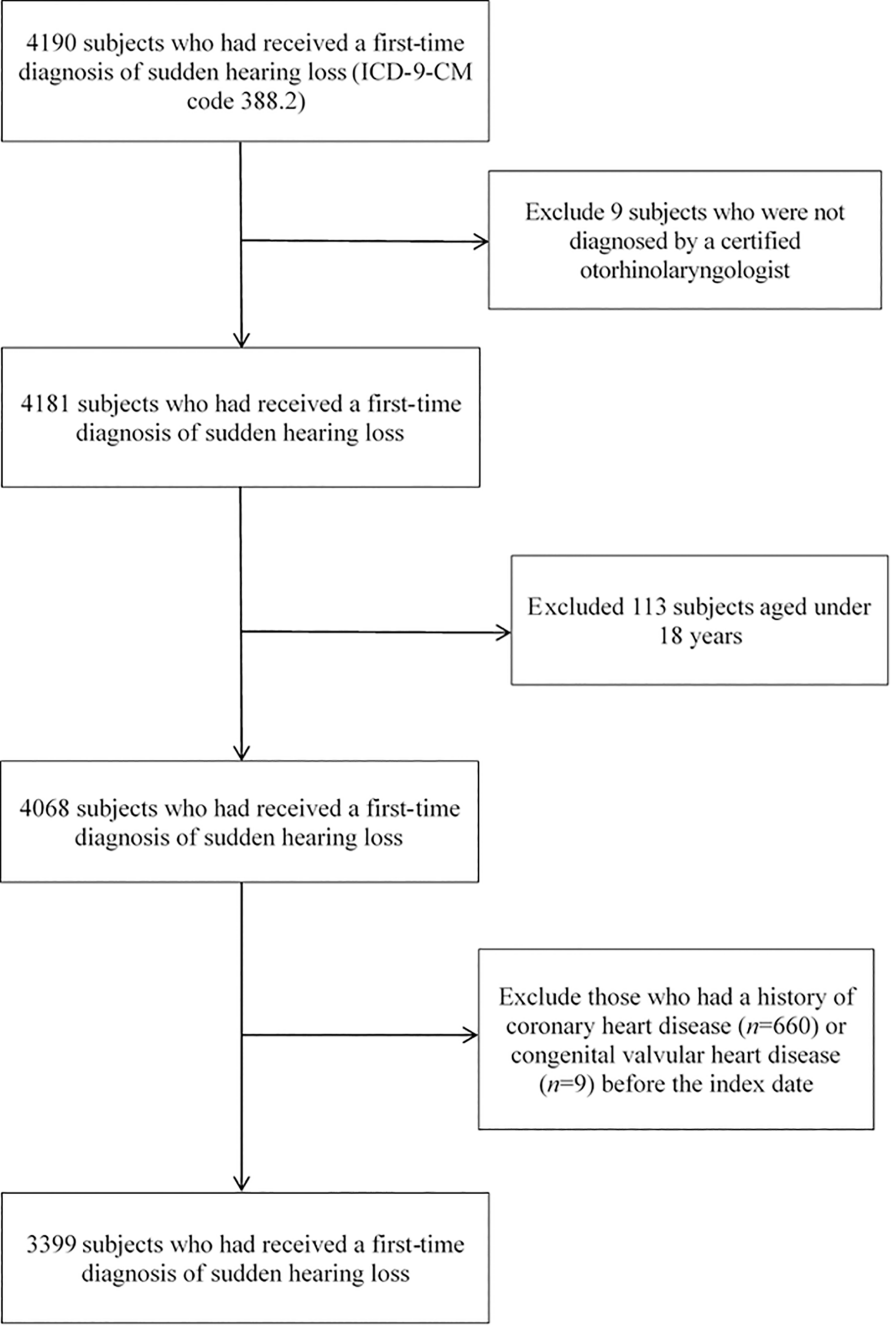
The selection procedures of cases.

As for the selection of cases, we first selected 4190 subjects who had received a first-time diagnosis of sudden hearing loss (ICD-9-CM code 388.2) during ambulatory care visits from January 2005 to December 2013. We assigned the date of receiving the first SSNHL diagnosis as the index date for cases. In order to increase the diagnostic validity of this administrative dataset, we only included 4181 SSNHL cases who were diagnosed by a certified otorhinolaryngologist. We further excluded 113 patients aged under 18 years. We also excluded those who had a history of coronary heart disease (ICD-9-CM codes 410–414 or 429.2) (*n* = 660) or congenital valvular heart disease (VHD) (ICD-9-CM code 746 or 746.x) (*n* = 9) before the index date. As a result, 3399 patients with SSNHL were included as cases. The selection procedures were shown in Fig 1.

The controls were also retrieved from the remaining beneficiaries of the LHID2005. We first excluded patients with any claim showing a diagnosis of SSNHL, coronary heart disease, or congenital VHD since initiation of the NHI program in 1995. We then used propensity score matching to select 3399 comparison patients (one for every case) from the Registry of Beneficiaries of the LHID2005. The selected matching variables included age, sex, monthly income (NT$0~15,840, NT$15,841~25,000, ≥NT$25,001; the average exchange rate in 2013 was US$1.00≈New Taiwan (NT)$30), geographical location (northern, central, southern and eastern), urbanization level of the patient’s residence, the year of the index date, hypertension, diabetes, and hyperlipidemia. For cases, the year of the index date was the year in which they received their first SSNHL diagnosis. However, for controls, the year of the index date was simply a matched year in which the controls had a medical utilization. We also assigned the date of their first use of ambulatory care occurring during that matched year as the index date for controls. In addition, the reasons for matching cases and controls using the variables of urbanization level, monthly income and geographic region was help assure that cases and controls were reasonably similar with regard to unmeasured neighborhood socioeconomic characteristics. As a result, this study included 3399 cases and 3399 controls.

### Exposure assessment

This study identified MVP cases by the diagnosis of ICD-9-CM code 424.0 in a medical claim during an ambulatory care visit. We only included those subjects who were diagnosed by a certified cardiologist in order to increase the diagnostic validity. Additionally, we only included those MVP cases with at least one diagnosis of MVP before the index date.

### Statistical analysis

All analyses were performed using the SAS system (SAS System for Windows, vers. 8.2, SAS Institute, Cary, NC). This study used Chi-squared tests to compare differences in sociodemographic characteristics including monthly income, geographic location and urbanization level of the patient’s residence, and medical comorbidities between cases and controls. We also performed conditional logistic regression analyses conditioned on gender, age, monthly income, urbanization level, geographic region, hyperlipidemia, diabetes, and hypertension to examine the association of SSNHL with previously diagnosed MVP. The conventional *p*≤0.05 was adopted to assess statistical significance.

## Results and discussion

Table 1 shows the distributions of sociodemographic characteristics and medical comorbidities between cases and controls. Of the 6798 sampled subjects, the mean age was 49.2 years with a standard deviation (SD) of 16.1 years. There was no significant difference between cases and controls in terms of age, sex, monthly income, urbanization level, geographic region, or the prevalences of hyperlipidemia, diabetes, and hypertension.

**Table 1:** Demographic characteristics of patients with sudden sensorineural hearing loss and controls in Taiwan (n = 6798).

Variable	Patients with sudden sensorineural hearing loss (*n* = 3399)		Controls (*n* = 3399)		*p* value
	Total no.	Percent	Total no.	Percent	
Males	1814	53.4	1814	53.4	>0.999
Age, mean (SD), years	49.2 (16.1)		49.2 (16.1)		>0.999
Monthly income					>0.999
<NT$1~15,841	1277	37.6	1277	37.6	
NT$15,841~25,000	1150	33.8	1150	33.8	
≥NT$25,001	972	28.6	972	28.6	
Geographic region					0.993
Northern	1596	47.0	1592	46.8	
Central	856	25.2	862	25.4	
Eastern	875	25.7	876	25.8	
Southern	72	2.1	69	2.0	
Urbanization level					0.621
1 (most urbanized)	1032	30.4	1082	31.8	
2	1020	30.0	995	29.3	
3	536	15.8	516	15.2	
4	456	13.4	436	12.8	
5 (least urbanized)	355	10.4	370	10.9	
Hyperlipidemia	553	16.3	553	16.3	>0.999
Diabetes	438	12.9	438	12.9	>0.999
Hypertension	756	22.2	756	22.2	>0.999

*Note*: In 2013, the average exchange rate was US$1≈New Taiwan (NT)$30.

SD, standard deviation.

Prevalences of prior MVP of cases and controls are shown in Table 2. In total, 131 (1.93%) sampled patients had received a diagnosis of MVP prior to the index date. A Chi-squared test further revealed that there was a significant difference in the prevalence of prior MVP between cases and controls (2.41% vs. 1.44%, *p =* 0.004). The conditional logistic regression analysis also suggested that the odds ratio (OR) of prior MVP for cases was 1.69 (95% confidence interval (CI): 1.18~2.42) compared to controls.

**Table 2:** Prevalence, crude odds ratios (ORs), and 95% confidence intervals (CIs) for mitral valve prolapse among sampled subjects.

Presence of mitral valve prolapse	Total (*n* = 6798)		Patients with sudden sensorineural hearing loss (*n* = 3399)		Controls (*n* = 3399)	
	*n*, Percent		*n*, Percent		*n*, Percent	
Yes	131	1.93	82	2.41	49	1.44
No	6667	98.07	3317	97.59	3350	98.56
OR (95% CI)	—		1.69** (1.18~2.42)		1.00	

*Notes*: *Notes*: The OR was calculated by a logistic regression which was conditioned on gender, age, monthly income, urbanization level, geographic region, hyperlipidemia, diabetes, and hypertension.

** p<0.01.

To the best of our knowledge, this is the first large-scale retrospective study to look at the possible relationship between MVP and SSNHL. Our results revealed that MVP was significantly associated with SSNHL. A conditional logistic regression indicated that the OR for SSNHL among patients with MVP was 1.69 (95% CI = 1.18~2.42) after taking demographic variables and comorbidities into considerations.

Our findings agree with related uncontrolled studies by Solanella et al. and Moulonguet et al., who reported that the majority of study subjects examined with 2-D echocardiography for sudden hearing loss had MVP[4, 6] The speculation was later affirmed by a larger controlled prospective study by Vazquez et al[5]. Their results showed that the rate of MVP was remarkably higher among patients with SSNHL (29.1%~67%), implying a possible association between MVP and SSNHL.

MVP was previously thought to be benign in behavior; however, emerging evidence has shown that MVP may also lead to some complications, including mitral regurgitation, atrial and ventricular remodeling, heart arrhythmias, infectious endocarditis, sudden death, and most notably, thromboembolic events, such as cerebral ischemia and amaurosis fugax. Two decades ago, platelet activity was studied among patients with MVP by Walsh et al., and they observed platelet hyperactivity compared to a control group, suggesting the platelets may play a vital role in the pathogenesis of thromboembolisms in MVP[7]. This notion was buttressed by Icli et al., whose findings showed that the mean platelet volume is elevated in MVP patients and is more reactive[8]. Those studies indicated that MVP patients are particularly vulnerable to certain thromboembolic events which can consequently endanger oxygen-sensitive organs, such as the retina, brain, and cochlea.

The underlying mechanism of the association between MVP and SSNHL is likely related to thromboembolic events. MVP is primarily characterized by myxomatous degeneration of mitral valve leaflets, which substantially disrupts the normal mitral endothelium. The deteriorated mitral endothelium is susceptible to thromboses and bacterial infections. Previous studies demonstrated the occurrence of thromboembolic events in patients with MVP, including ischemic neurologic or retinal emobli issues[9–11]. Kostuk et al. suggested that the disrupted mitral endothelium serves as a nidus for thrombus formation, which later predisposes the patient to cerebral ischemic events. Those studies are congruent with the etiology behind these embolic events have a cardiac origin[12].

The cochlea, situated within the petrous part of the temporal bone, is devoid of collateral vasculature; therefore, it is highly vulnerable to ischemia [13, 14]. Supported by temporal bone and animal studies, cochlear blood flow reduction caused by the thromboembolic events induce decreased oxygen supply and resulting oxidative stress, leading to hair cell or ganglion cell damage and therefore hearing loss[15–18]. Recent molecular and genetic studies uncovered several signaling pathways involved in the pathogenesis of MVP. Emerging evidence suggests that mutations in the FBN1 gene, which encode structural extracellular matrix (ECM) proteins, lead to myxomatous degeneration of mitral valve leaflets[19–22]. The accumulation of myxoid ECM is the cause of increased transforming growth factor (TGF)-β activation and signaling, and as TGF signaling is a key regulator of both inflammatory and immune responses in the cochlea[23], increased TGF-β signaling in MVP patients may also play a significant role in the pathogenesis of SSHNL.

Several limitations need to be acknowledged in the present study. First, the number of MVP patients could have been underrated, as asymptomatic patients with mild MVP were under-recognized. However, although there was no apparent reason to consider that asymptomatic patients with mild MVP afterwards would distinctively present between cases and controls, the association between MVP and SSNHL should be interpreted with extreme caution. Second, the current study obtained database from NHI only offer secondary dataset and does not include original medical record. The lack of audiometric data and the severity of MVP and SSNHL could possibly compromise our findings. Although epidemiologic evidence suggests the association, replication of studies to validate MVP as a risk factor for SSNHL is imperative. Third, there is a possibility of misclassification. Non-differential misclassification might have biased the results toward the null hypothesis. Fourth, our study population mainly consisted of ethnic Chinese, i.e., Taiwanese. As the distribution of MVP may differ between geographic regions, further validation of our results in other ethnic groups may be required.

## Conclusions

Our study found that there was an association between MPV and SSNHL in the general population. Although the difference in the prevalence of MPV between cases and controls was small, the results call for more awareness of the possible concurrence of SSNHL among physicians and patients with MPV. Further clinico-epidemiological studies are warranted to confirm the findings in other regions and races.
